# Unprecedented Diversity of ssDNA Phages from the Family *Microviridae* Detected within the Gut of a Protochordate Model Organism (*Ciona robusta*)

**DOI:** 10.3390/v10080404

**Published:** 2018-07-31

**Authors:** Alexandria Creasy, Karyna Rosario, Brittany A. Leigh, Larry J. Dishaw, Mya Breitbart

**Affiliations:** 1 College of Marine Science, University of South Florida, St. Petersburg, FL 33701, USA; creasy@mail.usf.edu (A.C.); krosari2@mail.usf.edu (K.R.), bleigh@mail.usf.edu or brittany.a.leigh@vanderbilt.edu (B.A.L.); 2 Department of Pediatrics, Children’s Research Institute, University of South Florida, St. Petersburg, FL 33701, USA

**Keywords:** phage, ssDNA, *Microviridae*, virome, microbiome, gut, *Ciona*, invertebrate

## Abstract

Phages (viruses that infect bacteria) play important roles in the gut ecosystem through infection of bacterial hosts, yet the gut virome remains poorly characterized. Mammalian gut viromes are dominated by double-stranded DNA (dsDNA) phages belonging to the order *Caudovirales* and single-stranded DNA (ssDNA) phages belonging to the family *Microviridae*. Since the relative proportion of each of these phage groups appears to correlate with age and health status in humans, it is critical to understand both ssDNA and dsDNA phages in the gut. Building upon prior research describing dsDNA viruses in the gut of *Ciona robusta*, a marine invertebrate model system used to study gut microbial interactions, this study investigated ssDNA phages found in the *Ciona* gut. We identified 258 *Microviridae* genomes, which were dominated by novel members of the *Gokushovirinae* subfamily, but also represented several proposed phylogenetic groups (Alpavirinae, Aravirinae, Group D, Parabacteroides prophages, and Pequeñovirus) and a novel group. Comparative analyses between *Ciona* specimens with full and cleared guts, as well as the surrounding water, indicated that *Ciona* retains a distinct and highly diverse community of ssDNA phages. This study significantly expands the known diversity within the *Microviridae* family and demonstrates the promise of *Ciona* as a model system for investigating their role in animal health.

## 1. Introduction

Recent studies of host-microbe interactions have recognized the importance of the holobiont, which acknowledges the complex partnerships between an animal and the entirety of its associated microbial communities [1]. The complex, dynamic relationship between an animal host and its microbial associates impacts many aspects of host physiology, including the procurement of nutrients and metabolic output [2,3]. In the nutrient- and mucus-rich gut of animals, the bacterial component of the microbiome can contribute orders of magnitude more gene products to host physiology than the host genome itself [4]. Although significant attention has been given to the metabolic contributions from the cellular component of the microbiome (i.e., bacteria, archaea, and fungi), it is now recognized that viruses also play important roles in the holobiont, both directly through infecting animal host cells, and indirectly through mediating microbiome dynamics.

Viruses present in gut communities are dominated by phages, which are viruses that infect bacteria [5,6,7,8,9,10,11]. The abundances of phages and bacteria are approximately equal in the human intestine [6,7,12], which contrasts with ratios observed in environmental samples where phages are typically ten times as abundant as bacteria [13]. Since phages can have dramatic influences on the structure and function of bacterial communities through host-specific lysis and horizontal gene transfer [13,14,15], phage-bacterial interactions appear crucial in all environments studied to date [15,16,17,18,19], including communities that are found in close association with animals [4,20,21,22,23,24]. While phage dynamics likely have dramatic influences on the physiology of the animal host, very little is known about the role of viromes (the cumulative viral community associated with a given host) or their impacts within the gut environment [6,8,9,10,25,26,27,28,29,30].

A variety of factors may influence the abundance, diversity, and ecological roles of phages in a complex ecosystem like the gut [31,32]. However, characterization of phage community composition is difficult due to a lack of universal gene markers within phage genomes [16]. Viral metagenomics, where the collective viral nucleic acids from a given sample are sequenced, is an efficient alternative approach for exploring the gut virome [5,12]. Although both single-stranded DNA (ssDNA) and double-stranded DNA (dsDNA) phages have been identified in animal guts [27,33], the diversity of dsDNA phages remains the best characterized due to their large representation in culture collections and genomic sequence databases. In addition, standardly used library construction kits (e.g., the NexteraXT DNA library preparation kit, Illumina, San Diego, CA, USA) and viromic studies using linker-amplified shotgun sequencing approaches bias against ssDNA viruses [34]. However, the implementation of rolling circle amplification (RCA) to obtain enough DNA for sequencing has revealed a large diversity of genomes from ssDNA viruses in the guts of humans and other animals [6,7,12,33,35,36,37,38,39,40,41,42,43,44,45,46,47].

Studies investigating microbial communities in the human gut have shown that the microbiome structure seen in healthy newborns develops and changes dramatically within the first 2–3 years of life from a nearly sterile gut environment to a dynamic community maintained throughout adulthood [27]. Human gut phage communities are dominated by dsDNA phages from the order *Caudovirales* and members of the ssDNA phage family *Microviridae* [27,48,49]. Infant viromes appear to contain the highest phage richness at birth, which decreases with age [27,50]. While *Caudovirales* dominate in the earliest samples, the composition of the virome changes over time, with an increased relative abundance and richness of *Microviridae* by 2 years of age [27]. Gut phage communities often change in composition during disease states, such as inflammatory bowel disease, where the *Microviridae*:*Caudovirales* ratio decreases [51]. Despite the fact that several recent studies have highlighted phages belonging to the *Microviridae* as important gut virome components associated with an individual’s health state [12,27,33,37,51,52], little is known about the roles and dynamics of *Microviridae* in this complex ecosystem.

Studies leveraging simpler model systems to study the gut virome can enable hypothesis-driven experimental approaches to dissect these multifaceted biological and ecological processes. We have been developing a marine cosmopolitan sea squirt species, *Ciona robusta* (formerly *Ciona intestinalis* subtype A), in efforts to interrogate gut microbiome dynamics. This sessile, invertebrate chordate is a well-studied developmental model [53] with a sequenced genome [54]. Because *Ciona* is a filter feeding organism, its gut is a microcosm of microbial interactions that experience vast and continuous exposure to the large microbial and viral diversity found in seawater. Previous efforts have identified remarkable stability among some elements of the microbiome, which are distinct from surrounding seawater [20,55]. *Ciona* has structurally distinct gut compartments (stomach, midgut, hindgut) and maintains core bacterial species, some of which exhibit compartmentalization [20]. A recent effort characterizing the dsDNA *Ciona* gut virome revealed the dominance of tailed phages belonging to the order *Caudovirales*, as well as a diversity of eukaryotic dsDNA viruses. There was also evidence for compartmentalization of some components of the dsDNA virome, although to a lesser extent than seen for the corresponding bacterial communities [55]. Together, these findings suggest that strong selective pressures operate within the gut of this simple model organism. However, additional viral groups such as ssDNA viruses have yet to be characterized within *Ciona*, a finding that would support the use of this model system in studies to investigate the role of phages in animal guts.

The main objectives of the current study were to: (1) characterize the diversity of complete circular genomes related to the *Microviridae*, the most widely detected ssDNA phages among animals, from the *Ciona* gut, (2) evaluate if the identified ssDNA phages were unique to *Ciona* or if they were also found in water samples, and (3) determine if different gut compartments contained distinct ssDNA phage assemblages. We show that *Ciona* harbors a highly diverse community of ssDNA phages that is distinct from the water column and demonstrates some compartmentalization within the gut, but with the majority of genomes found in multiple gut compartments. The novel viral genomes reported here significantly broaden the known diversity of the *Microviridae* family, with a particular expansion in the subfamily *Gokushovirinae* and detection of a novel phylogenetic group.

## 2. Materials and Methods

### 2.1. Sample Collections and Library Preparations

Sequences described in this manuscript are derived from viromes generated by Leigh et al. (2018), which focused on analysis of dsDNA viral diversity. Animal guts were sampled from *Ciona robusta* harvested near San Diego, CA (M-Rep, Carlsbad, CA, USA). *Ciona robusta* is an invertebrate that is not regulated or protected by environmental agencies in the USA. M-Rep maintains a valid collection license through the state of California that allows them to ship to research institutions throughout the USA. Research at the University of South Florida was approved by biosafety protocols 1199 IA and 1351E. Upon sample arrival in Florida, ten animals were selected at random; five specimens were placed into virus-free 100 kD-filtered artificial seawater [56] to clear guts of dietary contents (water changed every 4 h for 24 h). These samples are referred to as ‘cleared guts.’ The remaining five animals were dissected with full gut contents. All animal guts, full (F) or cleared (C), were tri-sected (stomach (S), midgut (M), hindgut (H)) and snap-frozen in liquid nitrogen. Collected tissues from each gut type (*n* = 5) were disrupted in 3 mL of sterile suspension buffer using the GentleMACS dissociator (Miltenyi Biotec, Bergisch Gladbach, Germany). Tissue fragments were pelleted (6000× *g* for 10 min), the supernatant was filtered through a 0.22 μm pore size Sterivex filter (Merck Millipore, Burlington, MA, USA), and the filtrate containing virus-like particles (VLPs) was collected. In addition to the viral fraction from *Ciona* guts, viruses from surrounding seawater in Mission Bay (MB) where the animals were collected, as well as the flow-through holding-tank water (CB), were processed to determine if viruses detected in *Ciona* guts could be detected in the surrounding water. For this purpose, one liter of seawater was filtered through a 0.22 μm pore size Sterivex filter and VLPs in the filtrate were concentrated to 1 mL using a 100 kDa Amicon centrifugal filter (EMD, Merck Millipore). VLPs were purified and further concentrated via cesium chloride (CsCl) gradient centrifugation [56] and collecting the 1.2–1.5 g/mL fraction with a sterile syringe and needle into a 2 mL sterile tube. To remove potential bacteria or extracellular vesicles still present in the sample, chloroform (final concentration 20% *v*/*v*) was added to the viral fraction and incubated at room temperature for 10 min. Samples were then centrifuged for 30 s at maximum speed (20,000× *g*) and the top aqueous layer was recovered. Unencapsidated, free nucleic acids were then removed by treating with DNase I (2.5 U/μL final concentration) for 3 h at 37 °C with frequent vortexing; the nuclease was inactivated by treating with 20 mM final concentration of EDTA pH 8.0. Purified VLP samples were tested to rule out bacterial contamination by PCR amplification of the 16S rRNA gene using primers 27F and 1492R [57] and epifluorescence microscopy. Viral DNA was then extracted from 200 µL of the viral concentrate using the Qiagen MinElute Virus Spin Kit (Qiagen, Inc., Valencia, CA, USA) and amplified via rolling circle amplification (RCA) using the GenomiPhi V2 DNA Amplification Kit (GE Healthcare Life Sciences, Pittsburgh, PA, USA), resulting in ~1 μg DNA per sample. Three identical RCA reactions per sample were prepared and pooled for sequencing. Qubit (Thermo Fisher Scientific, Waltham, MA, USA) was used to determine the DNA concentration and amplification was verified with 1% agarose gel electrophoresis. Final, amplified products were cleaned via the MinElute PCR Purification Kit (Qiagen, Inc.). DNA quality and quantity were assessed using the BioAnalyzer 2100 (Agilent Technologies, Santa Clara, CA, USA). Sequencing was performed on an Illumina MiSeq platform generating mate-pair (2 × 250 bp) libraries (Operon, Eurofins MWG Operon LLC, Huntsville, AL, USA). Sequences were analyzed in the CyVerse Cyberinfrastructure using different bioinformatics applications (Apps) [58]. Briefly, raw sequences were trimmed based on quality scores using the Trimmomatic App version 0.35.0 [59] and quality-filtered sequences were then assembled using the SPAdes App version 3.6.0 [60]. Contigs were screened with the VirSorter App to detect potential viral sequences. VirSorter outputs were uploaded to MetaVir [61] (IDs 7811 (SF), 8143 (SC), 7815 (MF), 7814 (MC), 7812 (HF), 7910 (HC), 7816 (CB), 7819 (MB)).

### 2.2. Identification and Annotation of Microviridae Genomes

Since members of the *Microviridae* family have circular genomes, circular contig sequences were identified using MetaVir [62]. Circular contig sequences ranging from 1 kb to 8 kb in length were compared (BLASTx, *e*-value < 0.0001) against a curated database of 4120 *Microviridae* major capsid protein (MCP) amino acid sequences (Jonathan Vincent and Francois Enault, Université Clermont Auvergne). Contig sequences with significant matches in the *Microviridae* MCP database were then compared (BLASTx, *e*-value < 0.001) against the Genbank non-redundant (nr) database to eliminate contig sequences that had better matches to cellular organisms (i.e., false positives). BLASTx outputs were explored using the MEGAN community edition software v6.8.9 [63] to identify sequences related to the *Microviridae*. *Microviridae*-related sequences were then manually trimmed to unit length genomes by identifying repeated sequences, and unit-length genomes were annotated using Geneious v10.1.3 [64]. For this purpose, open reading frames (ORFs) encoding putative proteins >80 amino acids (aa) were compared against the Genbank nr database using BLASTp (*e*-value < 0.001). Whenever possible, ORFs were annotated based on phage proteins (PHA source database) found in the NCBI conserved domain database with nomenclature following Cherwa and Fane [65]. All genomes were manually edited to begin at the start codon of the MCP. Genome-wide and MCP pairwise identities were calculated using the sequence demarcation tool (SDT) v1.2 [66]. Genomes were clustered at 95% nucleic acid identity based on current species demarcation cutoffs for phages [67]. Complete genome sequences are available under Genbank Accession Numbers MH572269–MH572526.

### 2.3. Genome Comparisons and Phylogenetic Analysis

Reference MCP amino acid sequences, including VP1 (subfamily *Gokushovirinae*) and Protein F (subfamily *Bullavirinae*), were collected from GenBank. These reference sequences also contained select sequences related to *Microviridae* that were identified from metagenomes [37,68,69,70,71,72,73,74,75,76] and those integrated into bacterial genomes [36] (see Supplementary Table S1 for details). Reference MCP sequences were aligned with sequences identified in *Ciona* guts using MUSCLE [77] as implemented in Geneious v10.1.3 [64]. A maximum likelihood tree was then created using PhyML with aLRT-like probabilities for branch support [78] and visualized with FigTree v1.4.3 (http://tree.bio.ed.ac.uk/software/figtree/). Branches with probability values less than 0.70 were collapsed via TreeGraph 2 [79].

To evaluate the diversity of *Microviridae* in different *Ciona* gut compartments, a recruitment analysis was performed following the pipeline suggested for analysis of viral abundance and distribution through iVirus as implemented in the CyVerse Cyberinfrastructure [80]. For this purpose, Bowtiebatch v1.0.0 and Read2Ref v1.0.1 were used with default parameters to map reads from each gut compartment against the 258 *Microviridae* genomes, requiring reads to cover >75% of the genome to consider a genome present in a compartment [80]. A binary matrix (presence/absence) was used to assess the number of shared *Microviridae* genomes in the different compartments. This information was summarized using the Venn Diagram package [81] with community indexes visualized as a dendrogram using the Vegan package [82] created in R v3.3.2 [83]. The binary data was analyzed with three methods (Bray-Curtis index, Jaccard index, Euclidian dissimilarity), each resulting in congruent tree topologies. Note that the number of reads recruiting to a given *Microviridae* genome was not considered either in this study or in comparing these genomes to the dsDNA phages [55], since there are known biases created by RCA that lead to overrepresentation of ssDNA circular genomes [34,84,85].

## 3. Results

### 3.1. Diversity of Microviridae in Ciona Gut Compartments

Analysis of the *Ciona* gut viromes, which included six libraries representing viral sequences from three gut compartments (stomach, midgut, and hindgut) from cleared and full guts, revealed 488 circular contig sequences (1–8 kb in length) with BLAST similarity to members of the *Microviridae*. After clustering these genomes based on 95% genome-wide pairwise identity, a total of 258 genomes were identified and named with the prefix, *Ciona* gut microphage (CGM), followed by a number (Supplementary Table S2). The average size of the identified CGM genomes was ~4.3 kb with a range from 3.9 kb to 5.8 kb, which is consistent with previously described members of the family *Microviridae* [65,86]. Coverage of the genomes in the compartment from which they were originally assembled ranged from 2× to 1167×, with a mean coverage of 35× (Supplementary Table S2).

**Figure 1:**
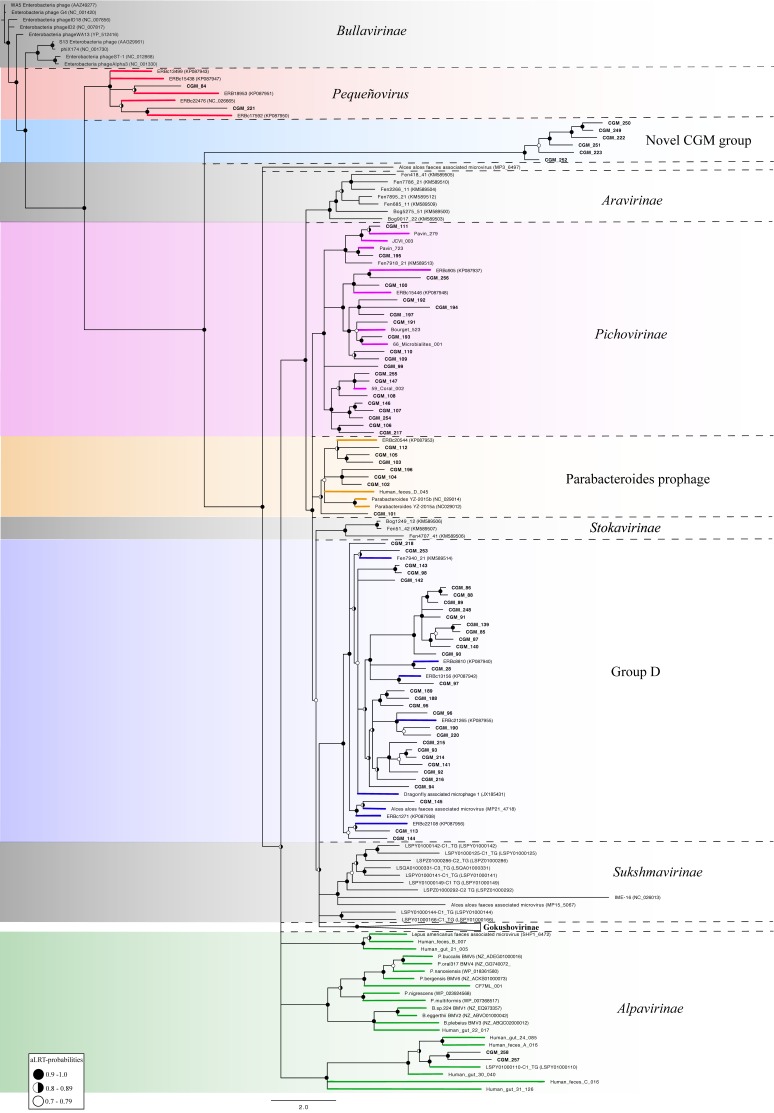
Maximum likelihood phylogenetic tree of predicted major capsid protein (MCP) sequences from the *Ciona* gut *Microviridae* (CGM, *n* = 258) along with representative sequences from previously proposed subfamilies (*n* = 96). The tree was created using PhyML with aLRT-probabilities; the scale bar represents the number of amino acid substitutions per site. Branches with probability values less than 0.7 were collapsed. Values greater than 0.7 are indicated at nodes. Suggested subfamily demarcations are delineated with dashed lines and colors based on previously classified sequences. Subfamilies for which CGM sequences were not identified are highlighted in grey color. Note: the *Gokushovirinae* sub-tree is displayed in Figure 2. Accession numbers for sequences used in this analysis are listed in Supplementary Tables S1 and S2.

**Figure 2:**
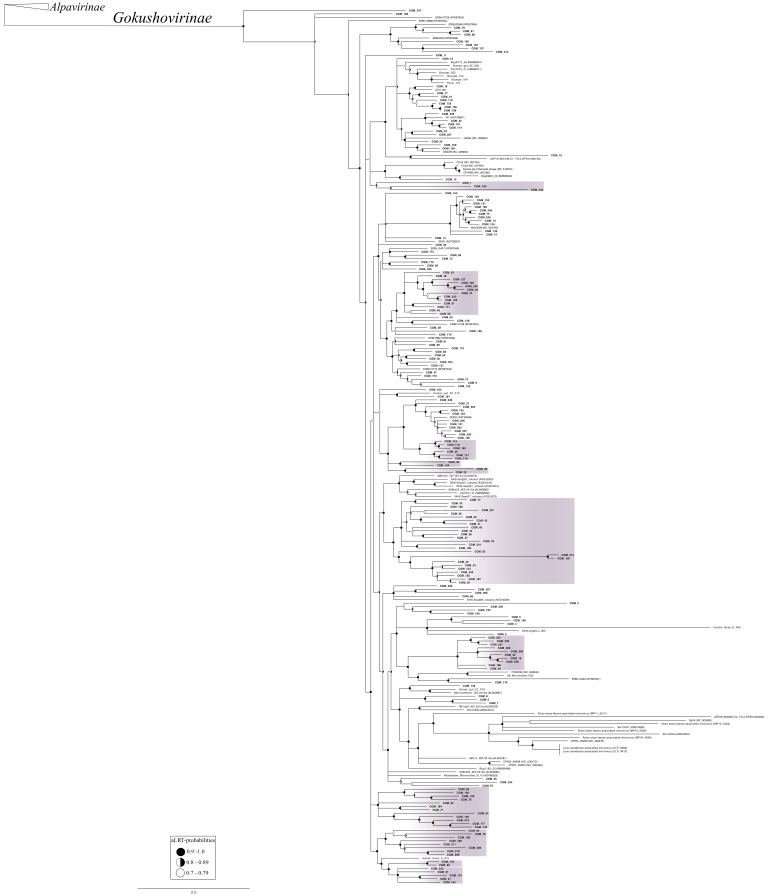
Maximum likelihood phylogenetic tree of predicted major capsid protein (MCP) sequences from the *Ciona* gut *Microviridae* (CGM, *n* = 188) that clustered within the established *Gokushovirinae* subfamily. MCP sequences representing Alpavirinae were used as an outgroup. The tree was created via PhyML with aLRT-probabilities; the scale bar represents the number of amino acid substitutions per site. Branches with probability values less than 0.7 were collapsed. Values greater than 0.7 are indicated at nodes. Clades highlighted in purple represent those where CGM sequences do not group with any previously described MCP sequences. Note: Accession numbers for sequences used in this analysis are listed in Supplementary Tables S1 and S2.

To assess diversity, each CGM genome was annotated and MCP amino acid sequences were used for phylogenetic analysis (Figure 1). Based on this analysis, the vast majority (*n* = 188) of CGM sequences grouped with the established subfamily *Gokushovirinae* [87], followed by sequences closely related to the proposed Group D microviruses (*n* = 33) [69] and the proposed subfamily Pichovirinae (*n* = 20) [73]. A smaller proportion of CGM sequences were related to Parabacteroides prophages (*n* = 7) [69,88], and members of the proposed Alpavirinae (*n* = 2) subfamily [36,37]. Six of the *Ciona* gut MCP sequences (CGM_251, CGM_223, CGM_252, CGM_222, CGM_249, CGM_250) form a distinct group from all previously described sequences and are referred to here as “Novel CGM Group”. None of the CGM MCP sequences grouped with the established *Bullavirinae* subfamily [87] or the proposed Aravirinae [68], Stokavirinae [68], or Sukshmavirinae [41] groups. However, two CGM sequences were most closely related to the pequeñoviruses, a proposed sister clade of the *Bullavirinae* [69]. Since 73% of the CGM genomes group within the *Gokushovirinae* subfamily, this subfamily is presented in a separate tree (Figure 2). Almost half (47%, *n* = 88) of the CGM MCP sequences within the *Gokushovirinae* subfamily do not group with previously reported sequences and share less than 70% aa identity with known *Gokushovirinae* MCP sequences. 

**Figure 3:**
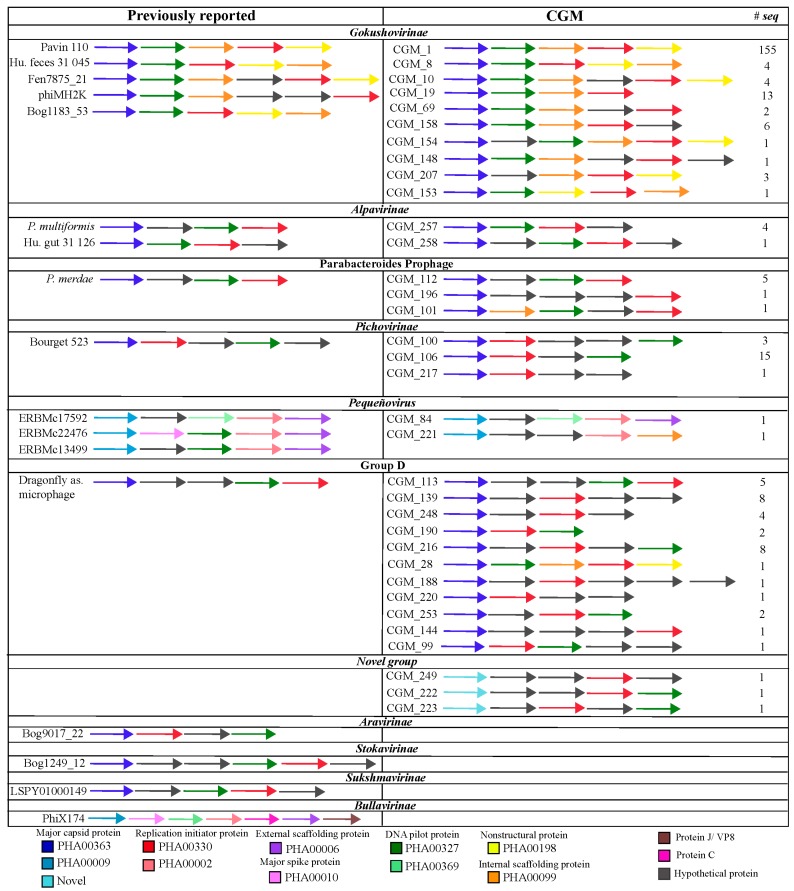
Gene synteny comparisons between previously described *Microviridae* genomes (left) and those discovered in the *Ciona* gut (right; CGM). All genomes were manually annotated to start at the major capsid protein (MCP) and open reading frames (ORFs) >80 aa are shown in linear fashion (i.e., overlapping genes are shown in order based on the position of the start codon). ORFs are color-coded based on PHA numbers (the phage protein subset of the Entrez protein cluster (PRK) database). One representative of each gene order known to exist within a given (proposed) subfamily is shown, and the numbers of CGM genomes containing a particular gene order are specified on the far right. The novel CGM group does not have representatives in the database, while the Aravirinae, Stokavirinae, Sukshmavirinae, and *Bullavirinae* were not detected among the CGM sequences. Details on the gene order for each CGM genome are available in Supplementary Table S2.

The gene synteny was compared between CGM genomes and previously reported *Microviridae* to evaluate if CGM genomes possess novel genome organizations (Figure 3). The CGM genomes expand upon previously identified gene organizations, yet many are syntenous with representative published sequences. The most diverse group, in terms of genome organization, was Group D, with 11 different patterns of gene organization.

### 3.2. Structure of Microviridae Communities

**Figure 4:**
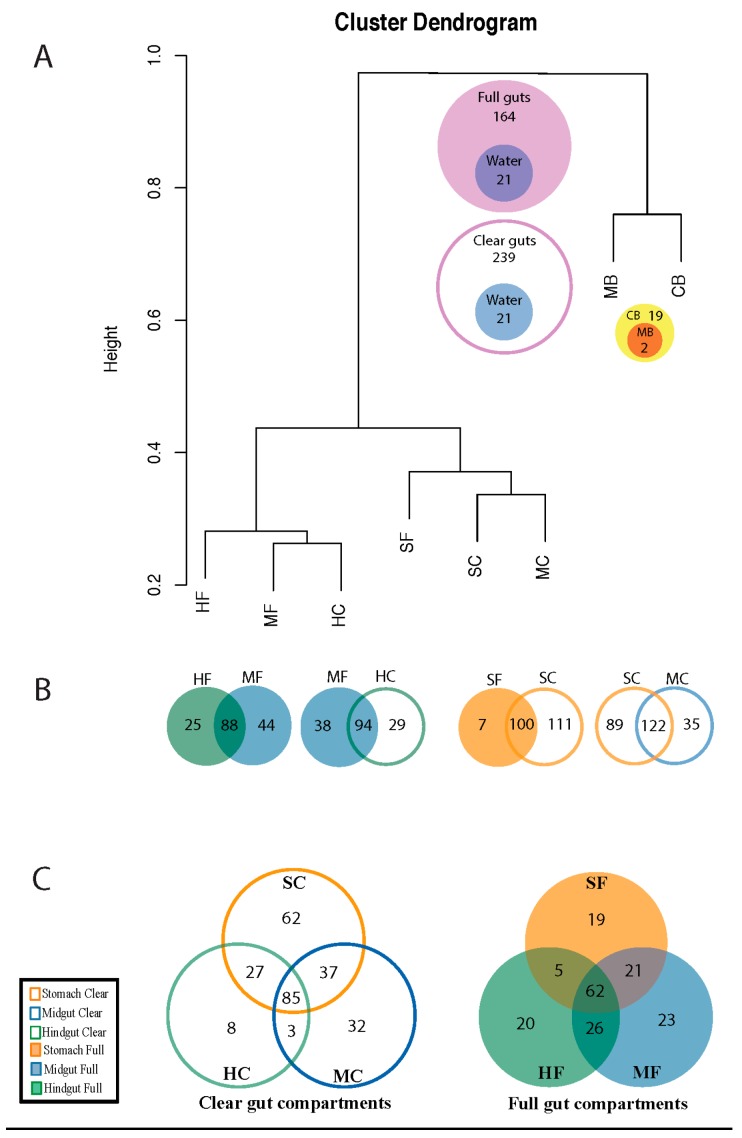
(**A**) Cluster dendrogram showing the relatedness among the CGM communities in the *Ciona* gut compartments and the surrounding water. (**B**) Venn diagrams showing comparisons between the closest groups on the dendrogram. (**C**) The three-way Venn diagrams specify shared and unique genomes detected in each of the compared groups. All diagrams were created based on the presence/absence of CGM genomes alone. The dendrogram was created using the Bray-Curtis dissimilarity index and the scale bar represents the dissimilarity values. Details on which CGM genomes were found in each compartment or water sample are available in Supplementary Table S2.

Richness of *Microviridae* genomes (i.e., the number of genomes that are <95% identical to each other) within gut compartments was compared among animals with either full or cleared guts. Stomach clear (SC) and midgut clear (MC) contained the highest number of genomes within the gut; these two compartments also share the largest number of genomes (Figure 4). Both water samples (MB & CB) group separately from the gut compartments (Figure 4), but all CGM sequences found in the water samples are also found throughout the gut compartments and all belong to the *Gokushovirinae* subgroup (Supplementary Table S2). Both the full and clear hindgut samples (HF and HC) share similarity with the midgut full (MF); however, the HF and MF share 88 genomes, the highest degree of overlap between the full gut compartments. The SC contained 211 *Microviridae* genomes; the largest number seen in any cleared gut compartment, while the HC had the lowest number of genomes (*n* = 123) of the cleared compartments, only 8 of which were unique to that compartment. Interestingly, despite being full of dietary material, the full gut compartments have a lower overall richness than the cleared ones. Four of the six sequences (CGM_251, CGM_223, CGM_250, CGM_257) belonging to the novel CGM group were only found in the hindgut full (Supplementary Table S2), and not seen in any other gut compartments. MF had the highest richness within the full gut, with a total of 132 genomes and 23 unique to that compartment. The lowest richness among the full compartments was found in the stomach, with 107 genomes and only 19 unique to SF.

## 4. Discussion

Most microbiome research to date has focused on bacterial communities, with descriptions of the virome only recently gaining traction [25,27,89]. Understanding the gut virome is relevant to both the host and the cellular microbiome because viruses, whether infecting eukaryotic, bacterial or archaeal hosts, can have profound influences in shaping gut homeostasis [18,26,90]. Here, we described ssDNA phages found in the *Ciona* gut in an effort to further characterize the virome of this invertebrate model organism.

Recent applications of RCA in virome studies has dramatically increased our discovery of small, ssDNA viruses including phages belonging to the family *Microviridae*. These phages have now been described in a variety of habitats, including non-human animal guts [40,41,42,44,45,46,71,74,91], human guts [6,7,12,27,33,35,38,39,49,92,93,94], reclaimed water [95], sewage [96,97], fresh water systems [73,85,96,98,99,100], marine systems [24,100,101,102,103,104,105,106,107,108], methane seeps [70], modern stromatolites [109], confined aquifers [110], sediments [76,85,108,111,112,113], dragonflies [72], and fruit trees [114]. Despite this rapid increase in sequence information for ssDNA phages, their identification in such diverse environments has yet to reveal information about their hosts or functions. Now that a diversity of phages related to the *Microviridae* has been identified in *Ciona*, future experiments using this model organism can aim to define the role of these viruses in an animal gut.

In characterizing the diversity of *Microviridae*, we found 258 viral genomes within the *Ciona* gut, vastly outnumbering the total number of *Microviridae* genomes reported from any single animal gut study [6,33,35,37,38,41,42,74,94]. Although previous studies frequently identify sequences related to the *Microviridae* (most often belonging to the subfamily *Gokushovirinae*), detailed analysis of the genomes is rarely reported and contig sequences are often not available. Based on analysis of the MCP, which is typically used as a phylogenetic marker for this phage group [37,68,96,109], we found a variety of novel *Microviridae* groups. The CGM MCP sequence diversity encompassed 6 of the 10 established or proposed *Microviridae* groups in the literature, and a novel CGM clade was identified (Figure 1). Sequences belonging to the *Gokushovirinae* dominated in the *Ciona* gut, with 47% of the CGM gokushovirus MCP sequences forming clades without representatives from the literature (Figure 2). This finding adds to the growing body of literature stressing the predominance of gokushoviruses throughout numerous environments [12,33,35,37,38,70,98,105,107].

Interestingly, the only definitive hosts for *Gokushovirinae* are obligate parasitic or predatory bacteria [65] and laboratory isolates infect *Bdellovibrio, Chlamydia,* and *Spiroplasma* [86]. Although the hosts for the gokushoviruses identified in this study are not known, it should be noted that 16S ribosomal RNA gene data from a previous study of the *Ciona* gut [20] found representatives from each of these three known bacterial host groups. In addition, it is possible that gokushoviruses infect other types of bacteria with intracellular life stages. Other potential hosts for the gokushoviruses are the symbiotic bacteria belonging to the genus *Endozoicomonas*, which are dominant members of the core *Ciona* microbiome [20] and commonly associated with tunicates and other marine invertebrates from which gokushoviruses are frequently detected [24,115,116]. Finally, it is notable that *Rickettsia* were prevalent in the animals processed for this study [55], serving as another possible obligate intracellular bacterial host for the identified gokushoviruses.

Many of the CGMs were found to share a significant degree of synteny with previously observed members of the *Microviridae* (Figure 3). However, unique genome organizations were observed for several of the CGM genomes. The largest number of unique genome organizations was noted for *Gokushovirinae* and Group D viruses. The genomic features distinguishing the two established *Microviridae* subfamilies (*Bullavirinae* and *Gokushovirinae*), namely the MCP and scaffolding proteins, were conserved [65,86]. Based on these features, the vast majority of the CGM genomes are consistent with the *Gokushovirinae* subfamily, with the exception being the two CGM genomes grouping with the *Bullavirinae* sister clade, Pequeñovirus, which have an external scaffolding protein [70].

Surprisingly, the large diversity of *Microviridae* observed in the *Ciona* gut was mostly absent from the surrounding seawater. The MB and CB water samples only contained 2 and 19 ssDNA phage genomes, respectively, all of which were present in the *Ciona* gut. The CB water originates from the holding tanks where the animals are placed between field collection and shipping. Though the animals spend less than 8 h in these waters, they are passing water through their siphons, feeding, and releasing feces, which could potentially contribute to the increased number of CGM genomes seen in this sample compared to MB, where the animals were originally collected. It is possible that some *Microviridae* virions are too small to be captured on a 100 kDa filter; however, the more likely explanation for the lack of overlap between the water and animal gut samples is that these viruses are less prevalent in seawater than the *Ciona* guts, especially if their hosts are intracellular bacteria. Nearly all of the CGM genomes (237 viral genomes) were unique to the *Ciona* gut (Figure 4), which parallels previous findings that dsDNA phages from the *Ciona* gut were also significantly different from those in the water column [55]. The fact that gokushovirus richness was much higher in the *Ciona* gut than in surrounding marine environments suggests that *Ciona* could be concentrating hosts for these phages, providing a model for further studies into their host range and infection dynamics.

No significant correlation was noted between taxonomic classification of *Microviridae* and gut compartmentalization in this study; diverse environmental factors may influence the structure of these systems, but none appear to influence how these phages are dispersed. However, distinct viral signatures are still found among the stomach, midgut and hindgut compartments, which can inform us of similarity among these niches and may provide clues as to how some of these specific viruses and/or their hosts are distributed. For example, while a large number of phages are predominately found in the stomach, the midgut clear compartment is more closely related to the stomach (clear and full) while the midgut full more closely resembles the hindgut (clear and full) (Figure 4). These findings suggest that the midgut is likely an intermediate reservoir of phages and that some level of compartmentalization exists among a portion of the viral communities.

Clearing of animals is a process used to void the gut of dietary and fecal material, but this process is inherently stressful to a filter feeder because food is restricted from their diet. This stress could, in part, account for the higher number of diverse viral types recovered from the SC, if the clearing process liberates viruses from the mucosal lining of the gut that otherwise would be under-sampled when the gut is full of dietary material. Retention seems to vary from the stomach to the hindgut as the *Microviridae* richness diminishes towards the most distal areas of the gut. This trend is not seen within the full compartments, where the rapid transit of dietary and fecal material through the gut likely impacts the distribution and/or compartmentalization of some viruses. For example, laboratory feeding experiments performed with fluorescently tagged food particles and/or bacteria in the Dishaw lab have revealed food pellets exiting the animals within 45 to 60 min after feeding. This rapid transit is hypothesized to impact the stability of some of these niches and likely diminishes compartmentalization of viral communities. The stress of clearing could also induce prophages and Leigh et al. [55] hypothesized that an increased prevalence of temperate phages may be due to prophage induction caused by the stress of clearing. Although originally thought to be strictly lytic, *Microviridae* sequences have been discovered as prophages within the genomes of *Bacteroidetes* and *Parabacteroides* species common in the human mouth and gut [36,69]. Seven CGM genomes with MCPs most similar to the Parabacteroides prophages were identified, suggesting the possibility that temperate ssDNA phages exist in this system. Although these sequences are in the minority in regards to the overall CGM diversity, it supports prior evidence that lysogeny is common in the *Ciona* gut [55].

This single study of the *Ciona* gut revealed more complete *Microviridae* genomes than any environmental [68,69,70,96,98,104,105,106,107] or gut study [6,27,33,37,38,39,41,42,94,117] to date. Many prior studies examining animal guts have defined the structure of the viral community but have not probed the diversity of ssDNA phages present. Studies reporting ssDNA phage genomes have identified low diversity in comparison to the CGM communities. As an example, one study examining the feces of patients with coronary heart disease found 12 *Gokushovirinae* genomes and 2 *Microviridae* genomes that did not group with any known subfamily [35]. Another study focusing on the guts of termites found 12 *Microviridae* genomes, 2 of which were *Gokushovirinae*, 3 that did not group with any reference genomes, and 7 that they proposed to belong to a new subfamily Sukshmavirinae [41]. In comparison with prior studies, the remarkable *Microviridae* diversity (258 genomes) described in this study originate from the guts of only 10 *Ciona* individuals. These findings suggest that these phages, some of which may be infecting intracellular bacteria, likely infect hosts that are concentrated in or colonize the *Ciona* gut. As a filter-feeding organism that concentrates organic material from seawater, *Ciona* provides unique opportunities to explore questions about *Microviridae* within the gut environment. This is particularly true as the *Ciona* microbiome [20] can be manipulated and tightly controlled by rearing the animals germ-free [118]. Juvenile *Ciona* are small enough that dozens to hundreds of transparent juveniles can be reared on small tissue culture dishes, facilitating experimental manipulations. Therefore, the *Ciona* system affords many opportunities to address hypothesis-driven questions, possibly resulting in development of the first model for understanding the host range and biology of the *Microviridae* in animal guts.

## Supplementary Materials

The following are available online at http://www.mdpi.com/1999-4915/10/8/404/s1, Table S1: Reference sequences used in constructing major capsid protein phylogenetic trees, Table S2: Details for each of the CGM phage genomes, including coverage, which compartment(s) they were detected in, and gene annotations.

## References

[1] Theis K.R., Dheilly N.M., Klassen J.L., Brucker R.M., Baines J.F., Bosch T.C., Cryan J.F., Gilbert S.F., Goodnight C.J., Lloyd E.A. (2016). Getting the hologenome concept right: An eco-evolutionary framework for hosts and their microbiomes. mSystems.

[2] Bordenstein S.R., Theis K.R. (2015). Host biology in light of the microbiome: Ten principles of holobionts and hologenomes. PLoS Biol..

[3] Nicholson J.K., Holmes E., Kinross J., Burcelin R., Gibson G., Jia W., Pettersson S. (2012). Host-gut microbiota metabolic interactions. Science.

[4] Turnbaugh P.J., Ley R.E., Hamady M., Fraser-Liggett C.M., Knight R., Gordon J.I. (2007). The human microbiome project. Nature.

[5] Breitbart M., Hewson I., Felts B., Mahaffy J.M., Nulton J., Salamon P., Rohwer F. (2003). Metagenomic analyses of an uncultured viral community from human feces. J. Bacteriol..

[6] Reyes A., Haynes M., Hanson N., Angly F.E., Heath A.C., Rohwer F., Gordon J.I. (2010). Viruses in the faecal microbiota of monozygotic twins and their mothers. Nature.

[7] Minot S., Sinha R., Chen J., Li H., Keilbaugh S.A., Wu G.D., Lewis J.D., Bushman F.D. (2011). The human gut virome: Inter-individual variation and dynamic response to diet. Genome Res..

[8] Mirzaei M.K., Maurice C.F. (2017). Ménage à trois in the human gut: Interactions between host, bacteria and phages. Nat. Rev. Microbiol..

[9] Manrique P., Dills M., Young M.J. (2017). The human gut phage community and its implications for health and disease. Viruses.

[10] Manrique P., Bolduc B., Walk S.T., van der Oost J., de Vos W.M., Young M.J. (2016). Healthy human gut phageome. Proc. Natl. Acad. Sci. USA.

[11] Yutin N., Makarova K.S., Gussow A.B., Krupovic M., Segall A., Edwards R.A., Koonin E.V. (2018). Discovery of an expansive bacteriophage family that includes the most abundant viruses from the human gut. Nat. Microbiol..

[12] Kim M.-S., Park E.-J., Roh S.W., Bae J.-W. (2011). Diversity and abundance of single-stranded DNA viruses in human feces. Appl. Environ. Microbiol..

[13] Suttle C.A. (2007). Marine viruses—Major players in the global ecosystem. Nat. Rev. Microbiol..

[14] Koskella B., Brockhurst M.A. (2014). Bacteria–phage coevolution as a driver of ecological and evolutionary processes in microbial communities. FEMS Microbiol. Rev..

[15] Shapiro O.H., Kushmaro A., Brenner A. (2010). Bacteriophage predation regulates microbial abundance and diversity in a full-scale bioreactor treating industrial wastewater. ISME J..

[16] Rohwer F., Thurber R.V. (2009). Viruses manipulate the marine environment. Nature.

[17] Breitbart M. (2012). Marine viruses: Truth or dare. Annu. Rev. Mar. Sci..

[18] Scarpellini E., Ianiro G., Attili F., Bassanelli C., De Santis A., Gasbarrini A. (2015). The human gut microbiota and virome: Potential therapeutic implications. Dig. Liver Dis..

[19] Thurber R.V., Payet J.P., Thurber A.R., Correa A.M. (2017). Virus–host interactions and their roles in coral reef health and disease. Nat. Rev. Microbiol..

[20] Dishaw L.J., Flores-Torres J., Lax S., Gemayel K., Leigh B., Melillo D., Mueller M.G., Natale L., Zucchetti I., De Santis R. (2014). The gut of geographically disparate *Ciona intestinalis* harbors a core microbiota. PLoS ONE.

[21] Sanders J.G., Beichman A.C., Roman J., Scott J.J., Emerson D., McCarthy J.J., Girguis P.R. (2015). Baleen whales host a unique gut microbiome with similarities to both carnivores and herbivores. Nat. Commun..

[22] Soverini M., Quercia S., Biancani B., Furlati S., Turroni S., Biagi E., Consolandi C., Peano C., Severgnini M., Rampelli S. (2016). The bottlenose dolphin (*Tursiops truncatus*) faecal microbiota. FEMS Microbiol. Ecol..

[23] Isaacson R., Kim H.B. (2012). The intestinal microbiome of the pig. Anim. Health Res. Rev..

[24] Laffy P.W., Wood-Charlson E.M., Turaev D., Jutz S., Pascelli C., Botté E.S., Bell S.C., Peirce T.E., Weynberg K.D., van Oppen M.J. (2018). Reef invertebrate viromics: Diversity, host specificity and functional capacity. Environ. Microbiol..

[25] Ogilvie L.A., Jones B.V. (2015). The human gut virome: A multifaceted majority. Front. Microbiol..

[26] Carding S.R., Davis N., Hoyles L. (2017). The human intestinal virome in health and disease. Aliment. Pharmacol. Ther..

[27] Lim E.S., Zhou Y., Zhao G., Bauer I.K., Droit L., Ndao I.M., Warner B.B., Tarr P.I., Wang D., Holtz L.R. (2015). Early life dynamics of the human gut virome and bacterial microbiome in infants. Nat. Med..

[28] Mills S., Shanahan F., Stanton C., Hill C., Coffey A., Ross R.P. (2013). Movers and shakers: Influence of bacteriophages in shaping the mammalian gut microbiota. Gut Microbes.

[29] Metzger R.N., Krug A.B., Eisenächer K. (2018). Enteric virome sensing—Its role in intestinal homeostasis and immunity. Viruses.

[30] Keen E.C., Dantas G. (2018). Close encounters of three kinds: Bacteriophages, commensal bacteria, and host immunity. Trends Microbiol..

[31] Yatsunenko T., Rey F.E., Manary M.J., Trehan I., Dominguez-Bello M.G., Contreras M., Magris M., Hidalgo G., Baldassano R.N., Anokhin A.P. (2012). Human gut microbiome viewed across age and geography. Nature.

[32] O’Toole P.W., Jeffery I.B. (2015). Gut microbiota and aging. Science.

[33] Reyes A., Blanton L.V., Cao S., Zhao G., Manary M., Trehan I., Smith M.I., Wang D., Virgin H.W., Rohwer F. (2015). Gut DNA viromes of Malawian twins discordant for severe acute malnutrition. Proc. Natl. Acad. Sci. USA.

[34] Roux S., Solonenko N.E., Dang V.T., Poulos B.T., Schwenck S.M., Goldsmith D.B., Coleman M.L., Breitbart M., Sullivan M.B. (2016). Towards quantitative viromics for both double-stranded and single-stranded DNA viruses. PeerJ.

[35] Guo L., Hua X., Zhang W., Yang S., Shen Q., Hu H., Li J., Liu Z., Wang X., Wang H. (2017). Viral metagenomics analysis of feces from coronary heart disease patients reveals the genetic diversity of the *Microviridae*. Virol. Sin..

[36] Krupovic M., Forterre P. (2011). *Microviridae* goes temperate: Microvirus-related proviruses reside in the genomes of *Bacteroidetes*. PLoS ONE.

[37] Roux S., Krupovic M., Poulet A., Debroas D., Enault F. (2012). Evolution and diversity of the *Microviridae* viral family through a collection of 81 new complete genomes assembled from virome reads. PLoS ONE.

[38] Waller A.S., Yamada T., Kristensen D.M., Kultima J.R., Sunagawa S., Koonin E.V., Bork P. (2014). Classification and quantification of bacteriophage taxa in human gut metagenomes. ISME J..

[39] Reyes A., Wu M., McNulty N.P., Rohwer F.L., Gordon J.I. (2013). Gnotobiotic mouse model of phage–bacterial host dynamics in the human gut. Proc. Natl. Acad. Sci. USA.

[40] Moreno P.S., Wagner J., Mansfield C.S., Stevens M., Gilkerson J.R., Kirkwood C.D. (2017). Characterisation of the canine faecal virome in healthy dogs and dogs with acute diarrhoea using shotgun metagenomics. PLoS ONE.

[41] Tikhe C.V., Husseneder C. (2018). Metavirome sequencing of the termite gut reveals the presence of an unexplored bacteriophage community. Front. Microbiol..

[42] D’arc M., Furtado C., Siqueira J.D., Seuánez H.N., Ayouba A., Peeters M., Soares M.A. (2018). Assessment of the gorilla gut virome in association with natural simian immunodeficiency virus infection. Retrovirology.

[43] Székely A.J., Breitbart M. (2016). Single-stranded DNA phages: From early molecular biology tools to recent revolutions in environmental microbiology. FEMS Microbiol. Lett..

[44] Zhang W., Li L., Deng X., Kapusinszky B., Pesavento P.A., Delwart E. (2014). Faecal virome of cats in an animal shelter. J. Gen. Virol..

[45] Smits S.L., Raj V.S., Oduber M.D., Schapendonk C.M., Bodewes R., Provacia L., Stittelaar K.J., Osterhaus A.D., Haagmans B.L. (2013). Metagenomic analysis of the ferret fecal viral flora. PLoS ONE.

[46] Li L., Shan T., Wang C., Côté C., Kolman J., Onions D., Gulland F.M., Delwart E. (2011). The fecal viral flora of California sea lions. J. Virol..

[47] Van den Brand J.M., van Leeuwen M., Schapendonk C.M., Simon J.H., Haagmans B.L., Osterhaus A.D., Smits S.L. (2011). Metagenomic analysis of the viral flora of pine marten and European badger feces. J. Virol..

[48] Minot S., Bryson A., Chehoud C., Wu G.D., Lewis J.D., Bushman F.D. (2013). Rapid evolution of the human gut virome. Proc. Natl. Acad. Sci. USA.

[49] Breitbart M., Haynes M., Kelley S., Angly F., Edwards R.A., Felts B., Mahaffy J.M., Mueller J., Nulton J., Rayhawk S. (2008). Viral diversity and dynamics in an infant gut. Res. Microbiol..

[50] Lim E.S., Wang D., Holtz L.R. (2016). The bacterial microbiome and virome milestones of infant development. Trends Microbiol..

[51] Norman J., Handley S., Baldridge M., Droit L., Liu C., Keller B., Kambal A., Monaco C., Zhao G., Fleshner P. (2015). Disease-specific alterations in the enteric virome in inflammatory bowel disease. Cell.

[52] Zuo T., Wong S.H., Lam K., Lui R., Cheung K., Tang W., Ching J.Y., Chan P.K., Chan M.C., Wu J.C. (2018). Bacteriophage transfer during faecal microbiota transplantation in *Clostridium difficile* infection is associated with treatment outcome. Gut.

[53] Satoh N., Satou Y., Davidson B., Levine M. (2003). *Ciona intestinalis*: An emerging model for whole-genome analyses. Trends Genet..

[54] Dehal P., Satou Y., Campbell R., Chapman J., Degnan B., De Tomaso A., Davidson B., Di Gregorio A., Gelpke M., Goodstein D. (2002). The draft genome of *Ciona intestinalis*: Insights into chordate and vertebrate origins. Science.

[55] Leigh B.A., Djurhuus A., Breitbart M., Dishaw L.J. (2018). The gut virome of the protochordate model organism, *Ciona intestinalis* subtype A. Virus Res..

[56] Thurber R.V., Haynes M., Breitbart M., Wegley L., Rohwer F. (2009). Laboratory procedures to generate viral metagenomes. Nat. Protoc..

[57] Weisburg W.G., Barns S.M., Pelletier D.A., Lane D.J. (1991). 16S ribosomal DNA amplification for phylogenetic study. J. Bacteriol..

[58] Goff S.A., Vaughn M., McKay S., Lyons E., Stapleton A.E., Gessler D., Matasci N., Wang L., Hanlon M., Lenards A. (2011). The iPlant collaborative: Cyberinfrastructure for plant biology. Front. Plant Sci..

[59] Bolger A.M., Lohse M., Usadel B. (2014). Trimmomatic: A flexible trimmer for Illumina sequence data. Bioinformatics.

[60] Bankevich A., Nurk S., Antipov D., Gurevich A.A., Dvorkin M., Kulikov A.S., Lesin V.M., Nikolenko S.I., Pham S., Prjibelski A.D. (2012). SPAdes: A new genome assembly algorithm and its applications to single-cell sequencing. J. Comput. Biol..

[61] Roux S., Tournayre J., Mahul A., Debroas D., Enault F. (2014). Metavir 2: New tools for viral metagenome comparison and assembled virome analysis. BMC Bioinform..

[62] Roux S., Faubladier M., Mahul A., Paulhe N., Bernard A., Debroas D., Enault F. (2011). Metavir: A web server dedicated to virome analysis. Bioinformatics.

[63] Huson D.H., Beier S., Flade I., Górska A., El-Hadidi M., Mitra S., Ruscheweyh H.-J., Tappu R. (2016). MEGAN community edition-interactive exploration and analysis of large-scale microbiome sequencing data. PLoS Comput. Biol..

[64] Kearse M., Moir R., Wilson A., Stones-Havas S., Cheung M., Sturrock S., Buxton S., Cooper A., Markowitz S., Duran C. (2012). Geneious Basic: An integrated and extendable desktop software platform for the organization and analysis of sequence data. Bioinformatics.

[65] Cherwa J.E., Fane B.A. (2001). *Microviridae*: Microviruses and Gokushoviruses. eLS.

[66] Muhire B.M., Varsani A., Martin D.P. (2014). SDT: A virus classification tool based on pairwise sequence alignment and identity calculation. PLoS ONE.

[67] Adriaenssens E., Brister J.R. (2017). How to name and classify your phage: An informal guide. Viruses.

[68] Labonté J.M., Suttle C.A. (2013). Previously unknown and highly divergent ssDNA viruses populate the oceans. ISME J..

[69] Quaiser A., Dufresne A., Ballaud F., Roux S., Zivanovic Y., Colombet J., Sime-Ngando T., Francez A.-J. (2015). Diversity and comparative genomics of *Microviridae* in *Sphagnum*-dominated peatlands. Front. Microbiol..

[70] Bryson S.J., Thurber A.R., Correa A., Orphan V.J., Vega Thurber R. (2015). A novel sister clade to the enterobacteria microviruses (family *Microviridae*) identified in methane seep sediments. Environ. Microbiol..

[71] Walters M., Bawuro M., Christopher A., Knight A., Kraberger S., Stainton D., Chapman H., Varsani A. (2017). Novel single-stranded DNA virus genomes recovered from chimpanzee feces sampled from the Mambilla Plateau in Nigeria. Genome Announc..

[72] Rosario K., Dayaram A., Marinov M., Ware J., Kraberger S., Stainton D., Breitbart M., Varsani A. (2012). Diverse circular ssDNA viruses discovered in dragonflies (Odonata: Epiprocta). J. Gen. Virol..

[73] Roux S., Enault F., Robin A., Ravet V., Personnic S., Theil S., Colombet J., Sime-Ngando T., Debroas D. (2012). Assessing the diversity and specificity of two freshwater viral communities through metagenomics. PLoS ONE.

[74] Kraberger S., Waits K., Ivan J., Newkirk E., VandeWoude S., Varsani A. (2018). Identification of circular single-stranded DNA viruses in faecal samples of Canada lynx (*Lynx canadensis*), moose (*Alces alces*) and snowshoe hare (*Lepus americanus*) inhabiting the Colorado San Juan Mountains. Infect. Genet. Evol..

[75] Shu P., Butt A.M., Mi Z., Wang W., An X., Pei G., Zhang Z., Huang Y., Zhang X., Shi T. (2015). Identification and genomic analysis of a novel member of *Microviridae*, IME-16, through high-throughput sequencing. Virol. Sin..

[76] Yu D.-T., He J.-Z., Zhang L.-M., Han L.-L. (2018). Viral metagenomics analysis and eight novel viral genomes identified from the Dushanzi mud volcanic soil in Xinjiang, China. J. Soils Sediments.

[77] Edgar R.C. (2004). MUSCLE: Multiple sequence alignment with high accuracy and high throughput. Nucleic Acids Res..

[78] Lefort V., Longueville J.-E., Gascuel O. (2017). SMS: Smart model selection in PhyML. Mol. Biol. Evol..

[79] Stöver B.C., Müller K.F. (2010). TreeGraph 2: Combining and visualizing evidence from different phylogenetic analyses. BMC Bioinform..

[80] Bolduc B., Youens-Clark K., Roux S., Hurwitz B.L., Sullivan M.B. (2017). iVirus: Facilitating new insights in viral ecology with software and community data sets imbedded in a cyberinfrastructure. ISME J..

[81] Chen H., Boutros P.C. (2011). VennDiagram: A package for the generation of highly-customizable Venn and Euler diagrams in R. BMC Bioinform..

[82] Oksanen J., Blanchet F.G., Kindt R., Legendre P. (2013). Vegan: Community Ecology.

[83] R Core Team (2014). R: A Language and Environment for Statistical Computing.

[84] Kim K.-H., Bae J.-W. (2011). Amplification methods bias metagenomic libraries of uncultured single-stranded and double-stranded DNA viruses. Appl. Environ. Microbiol..

[85] Kim K.-H., Chang H.-W., Nam Y.-D., Roh S.W., Kim M.-S., Sung Y., Jeon C.O., Oh H.-M., Bae J.-W. (2008). Amplification of uncultured single-stranded DNA viruses from rice paddy soil. Appl. Environ. Microbiol..

[86] Doore S.M., Fane B.A. (2016). The *Microviridae*: Diversity, assembly, and experimental evolution. Virology.

[87] Lefkowitz E.J., Dempsey D.M., Hendrickson R.C., Orton R.J., Siddell S.G., Smith D.B. (2018). Virus taxonomy: The database of the International Committee on Taxonomy of Viruses (ICTV). Nucleic Acids Res..

[88] Sakamoto M., Benno Y. (2006). Reclassification of Bacteroides distasonis, Bacteroides goldsteinii and Bacteroides merdae as Parabacteroides distasonis gen. nov., comb. nov., Parabacteroides goldsteinii comb. nov. and Parabacteroides merdae comb. nov.. Int. J. Syst. Evol. Microbiol..

[89] Reyes A., Semenkovich N.P., Whiteson K., Rohwer F., Gordon J.I. (2012). Going viral: Next-generation sequencing applied to phage populations in the human gut. Nat. Rev. Microbiol..

[90] Dahiya D., Renuka K. (2017). The gut virome: A neglected actor in colon cancer pathogenesis. Futur. Med..

[91] Jørgensen T.S., Xu Z., Hansen M.A., Sørensen S.J., Hansen L.H. (2014). Hundreds of circular novel plasmids and DNA elements identified in a rat cecum metamobilome. PLoS ONE.

[92] Zhang T., Breitbart M., Lee W.H., Run J.-Q., Wei C.L., Soh S.W.L., Hibberd M.L., Liu E.T., Rohwer F., Ruan Y. (2005). RNA viral community in human feces: Prevalence of plant pathogenic viruses. PLoS Biol..

[93] Santiago-Rodriguez T.M., Fornaciari G., Luciani S., Dowd S.E., Toranzos G.A., Marota I., Cano R.J. (2015). Natural mummification of the human gut preserves bacteriophage DNA. FEMS Microbiol. Lett..

[94] McCann A., Ryan F.J., Stockdale S.R., Dalmasso M., Blake T., Ryan C.A., Stanton C., Mills S., Ross P.R., Hill C. (2018). Viromes of one year old infants reveal the impact of birth mode on microbiome diversity. PeerJ.

[95] Rosario K., Nilsson C., Lim Y.W., Ruan Y., Breitbart M. (2009). Metagenomic analysis of viruses in reclaimed water. Environ. Microbiol..

[96] Hopkins M., Kailasan S., Cohen A., Roux S., Tucker K.P., Shevenell A., Agbandje-McKenna M., Breitbart M. (2014). Diversity of environmental single-stranded DNA phages revealed by PCR amplification of the partial major capsid protein. ISME J..

[97] Pearson V.M., Caudle S.B., Rokyta D.R. (2016). Viral recombination blurs taxonomic lines: Examination of single-stranded DNA viruses in a wastewater treatment plant. PeerJ.

[98] Zhong X., Guidoni B., Jacas L., Jacquet S. (2015). Structure and diversity of ssDNA *Microviridae* viruses in two peri-alpine lakes (Annecy and Bourget, France). Res. Microbiol..

[99] López-Bueno A., Tamames J., Velázquez D., Moya A., Quesada A., Alcamí A. (2009). High diversity of the viral community from an Antarctic lake. Science.

[100] Kim Y., Van Bonn W., Aw T.G., Rose J.B. (2017). Aquarium viromes: Viromes of human-managed aquatic systems. Front. Microbiol..

[101] Breitbart M., Salamon P., Andresen B., Mahaffy J.M., Segall A.M., Mead D., Azam F., Rohwer F. (2002). Genomic analysis of uncultured marine viral communities. Proc. Natl. Acad. Sci. USA.

[102] Angly F.E., Felts B., Breitbart M., Salamon P., Edwards R.A., Carlson C., Chan A.M., Haynes M., Kelley S., Liu H. (2006). The marine viromes of four oceanic regions. PLoS Biol..

[103] Bench S.R., Hanson T.E., Williamson K.E., Ghosh D., Radosovich M., Wang K., Wommack K.E. (2007). Metagenomic characterization of Chesapeake Bay virioplankton. Appl. Environ. Microbiol..

[104] Labonté J., Suttle C. (2013). Metagenomic and whole-genome analysis reveals new lineages of gokushoviruses and biogeographic separation in the sea. Front. Microbiol..

[105] Labonté J.M., Hallam S.J., Suttle C.A. (2015). Previously unknown evolutionary groups dominate the ssDNA gokushoviruses in oxic and anoxic waters of a coastal marine environment. Front. Microbiol..

[106] Tucker K.P., Parsons R., Symonds E.M., Breitbart M. (2011). Diversity and distribution of single-stranded DNA phages in the North Atlantic Ocean. ISME J..

[107] Yu D.-T., Han L.-L., Zhang L.-M., He J.-Z. (2018). Diversity and distribution characteristics of viruses in soils of a marine-terrestrial ecotone in East China. Microb. Ecol..

[108] Yoshida M., Mochizuki T., Urayama S.-I., Yoshida-Takashima Y., Nishi S., Hirai M., Nomaki H., Takaki Y., Nunoura T., Takai K. (2018). Quantitative viral community DNA analysis reveals the dominance of single-stranded DNA viruses in offshore upper bathyal sediment from Tohoku, Japan. Front. Microbiol..

[109] Desnues C., Rodriguez-Brito B., Rayhawk S., Kelley S., Tran T., Haynes M., Liu H., Furlan M., Wegley L., Chau B. (2008). Biodiversity and biogeography of phages in modern stromatolites and thrombolites. Nature.

[110] Smith R.J., Jeffries T.C., Roudnew B., Seymour J.R., Fitch A.J., Simons K.L., Speck P.G., Newton K., Brown M.H., Mitchell J.G. (2013). Confined aquifers as viral reservoirs. Environ. Microbiol. Rep..

[111] Yoshida M., Takaki Y., Eitoku M., Nunoura T., Takai K. (2013). Metagenomic analysis of viral communities in (hado) pelagic sediments. PLoS ONE.

[112] Han L.-L., Yu D.-T., Zhang L.-M., Shen J.-P., He J.-Z. (2017). Genetic and functional diversity of ubiquitous DNA viruses in selected Chinese agricultural soils. Sci. Rep..

[113] Reavy B., Swanson M.M., Cock P.J., Dawson L., Freitag T.E., Singh B.K., Torrance L., Mushegian A.R., Taliansky M. (2015). Distinct circular single-stranded DNA viruses exist in different soil types. Appl. Environ. Microbiol..

[114] Basso M.F., da Silva J.C.F., Fajardo T.V.M., Fontes E.P.B., Zerbini F.M. (2015). A novel, highly divergent ssDNA virus identified in Brazil infecting apple, pear and grapevine. Virus Res..

[115] Neave M.J., Apprill A., Ferrier-Pagès C., Voolstra C.R. (2016). Diversity and function of prevalent symbiotic marine bacteria in the genus *Endozoicomonas*. Appl. Microbiol. Biotechnol..

[116] Schreiber L., Kjeldsen K.U., Funch P., Jensen J., Obst M., López-Legentil S., Schramm A. (2016). *Endozoicomonas* are specific, facultative symbionts of sea squirts. Front. Microbiol..

[117] Krupovic M., Prangishvili D., Hendrix R.W., Bamford D.H. (2011). Genomics of bacterial and archaeal viruses: Dynamics within the prokaryotic virosphere. Microbiol. Mol. Biol. Rev..

[118] Leigh B.A., Liberti A., Dishaw L.J. (2016). Generation of germ-free *Ciona intestinalis* for studies of gut-microbe interactions. Front. Microbiol..

